# Use of autoantibodies against tumor-associated antigens as serum biomarkers for primary screening of cervical cancer

**DOI:** 10.18632/oncotarget.22231

**Published:** 2017-11-01

**Authors:** Yingji Jin, Seung Cheol Kim, Hyoung Jin Kim, Woong Ju, Yun Hwan Kim, Hong-Jin Kim

**Affiliations:** ^1^ Laboratory of Virology, College of Pharmacy, Chung-Ang University, Dongjak-Gu, Seoul 06974, South Korea; ^2^ Department of Obstetrics and Gynecology, Ewha Womans University College of Medicine, Yangcheon-Gu, Seoul 03760, South Korea

**Keywords:** cervical cancer, autoantibody, tumor associated antigen, enzyme-linked immunosorbent assay, cervical intraepithelial neoplasia

## Abstract

Serum autoantibodies against tumor-associated antigens (TAAs) have received much attention as potential biomarkers for early detection of cancers, since they can be detected in the early stages of cancers. Autoantibodies against Cancer Antigen 15-3 (CA15-3), carcinoembryonic antigen (CEA), Cancer Antigen 19-9 (CA19-9), c-Myc, p53, heat shock protein (Hsp)27 and Hsp70 have been suggested as potential markers for detecting several types of cancer. In the present study, the seven types of antibody listed above were evaluated for detecting cervical lesions. Enzyme-linked immunosorbent assays (ELISAs) were used to measure IgG levels of the autoantibodies in women with normal cytology, cervical intraepithelial neoplasia (CIN) I, CIN II, CIN III and cervical cancer. The increases of anti-CA15-3 and anti-CEA IgG in cervical cancer were more pronounced than the increases of the other markers, and the level of anti-CA19-9 IgG in CIN III stage was higher than in normal CIN I, CIN II or cervical cancer. A combination of ELISAs detecting anti-CA15-3, anti-CEA and anti-CA19-9 IgGs was found to reliably discriminate CINs from normal and to strongly differentiate cancer from normal (90.3% of sensitivity and 82.1% of specificity). We suggest that the combination of three ELISA may be useful for detecting cervical lesions.

## INTRODUCTION

Cervical cancer is the fourth most common cancer in women worldwide. GLOBOCAN estimates that 527,624 new cases were diagnosed as cervical cancer and 265,672 women died of it in 2012 [1]. Almost all cervical cancers are caused by infections with human papillomavirus (HPV) [2]. It is thought that invasive cervical cancer develops from a precancerous state termed cervical intraepithelial neoplasia (CIN) when infection with a high-risk HPV has persisted for 12-15 years [2]. CIN can be classified into CIN I, CIN II and CIN III according to the thickness of the layer of cervical epithelium containing abnormal cells [3]. Almost 90% of CIN I (low grade CIN) cases regress within two years whereas 5% of CIN II and 40% of CIN III cases (CIN II and CIN III are classified as high grade CINs) develop into invasive cervical cancer [4–6]. The 5-year survival rate of cervical cancer reaches 90% when it is detected early and treated appropriately [7]. However, survival at the late stage is just 15-35% [8]. Therefore, early detection of cervical cancer is the key to increasing survival and requires an effective primary screening system.

The Papanicolaou test (Pap test) has been used as the primary screening test for cervical cancer for over five decades and has contributed to decreasing both morbidity and mortality [9, 10]. However, its greatest drawback is its low sensitivity for detecting individuals at high risk of cervical cancer. It has been suggested that the sensitivity of a single Pap test for detecting CIN is only 51%, which results in missing patients at high risk of cervical cancer [11]. Therefore, the American College of Obstetricians and Gynecologists (ACOG) has recently recommended combining the HPV DNA test with the Pap test [12]. The HPV DNA test has high sensitivity but low specificity [13]. Actually 50% of patients with cervical cancer in the United States never attended primary screening for cervical cancer [14]. Therefore, developing a simpler and more accurate primary screening system for cervical cancer is important for overcoming low participation in the screening program. Serology tests are usually considered to be the simplest and most non-invasive tests and have the advantages that they allow high throughput screening, samples are easy to collect and examination is cost-effective.

Many researchers have focused on serum autoantibodies as biomarkers for cancer diagnosis. Proteins related to autoantibody responses can undergo overexpression, mutation, degradation or changes in glycosylation during carcinogenesis [15, 16]. The resulting aberrant proteins can elicit immune responses and are usually termed tumor associated antigens (TAAs) [16], and the serum autoantibodies are considered potential biomarkers for early detection of cancers [16].

At the same time there are several arguments against using autoantibodies as biomarkers. First, elevated anti-TAA antibody levels are found in only 10-30% of cancer patients [17]. Second, autoantibodies are not only expressed and amplified in cancers but also in other diseases [16], and finally some can also be detected in healthy individuals [16]. Therefore, the use of autoantibodies in cancer screening has created confusion when attempted on its own [16]. Recently, however, combination assays for several types of anti-TAA antibodies have shown significantly improved sensitivities and specificities [16]. Therefore, using autoantibodies as biomarkers for detecting cancers remains an attractive possibility.

Changes in the levels of autoantibodies against Cancer Antigen 15-3 (CA15-3), carcinoembryonic antigen (CEA), c-Myc, p53, heat shock protein (Hsp)27 and Hsp70 have been found in several types of cancer [18–26]. In addition, elevated levels of Cancer Antigen 19-9 (CA19-9) have been observed in lung, gastric, breast and pancreatic cancers as well as cervical cancer [27–31]. However there have been few studies investigating antibody responses against TAAs in cervical cancer.

The present study focused on serum antibodies against the TAAs CA15-3, CEA, CA19-9, c-Myc, p53, Hsp27 and Hsp70, which are involved in invasion, metastasis, progression, transformation and death of cancer cells [32]. The levels of autoantibodies against these seven TAAs were evaluated in women with normal cytology, CIN I, CIN II, CIN III and invasive cervical cancer, and their profiles were compared with those reported in other cancers.

## RESULTS

### Clinicopathological characteristics of the normal, CIN I, CIN II, CIN III and cervical cancer groups

The clinicopathological characteristics of the various cervical lesion groups are presented in Table 1. A total of 148 serum samples were collected, consisting of 28, 28, 30, 31 and 31 sera from healthy women and women with, CIN I, CIN II, CIN III and cervical cancer, respectively, and the mean ages of the corresponding patients were 45.6, 43.1, 45.9, 40.6 and 50.6 years, respectively. The proportion of squamous cell carcinomas in the cancer group was 77.4%, and that of adenocarcinomas was 19.4%. These proportions are virtually identical to those found generally (squamous cell carcinoma: 80%; adenocarcinoma: 20%) [33].

**Table 1:** Clinicopathological characteristics of normal, CIN I, CIN II, CIN III and cancer groups

	Normal (n=28)	CIN I (n=28)	CIN II (n=30)	CIN III (n=31)	Cancer (n=31)
**Age, years** (Mean ± SEM; range)^a^	45.6± 2.5 (20-79)	43.1± 2.2 (25-74)	45.9± 2.4 (28-75)	40.6± 2.0 (23-68)	50.6± 2.0 (31-74)
**Histology of cervical cancer** (Punch biopsy)					Squamous cell carcinoma (n=24; 77.4%) Adenocarcinoma (n=6; 19.4%) Adenosquamous carcinoma (n=1; 3.2%)
**Stage of cervical cancer^b^**					Ia (n=7) Ib (n=16) IIb (n=6) IVa (n=1) IVb (n=1)

Differences between groups in age were analyzed using two-tailed Student's *t*-test.

^a^ mean age of cancer group was higher than that of CIN I group or CIN III group (cancer vs CIN I, *p*=0.016; cancer vs CIN III, *p*=0.0009).

^b^ classified by International Federation of Obstetrics and Gynecology (FIGO) clinical staging system.

### Levels of autoantibodies (IgG) against the seven TAAs in the various sera

Enzyme-linked immunosorbent assays (ELISAs) were used to evaluate levels of circulating IgGs against the seven TAAs using the TAAs as coating antigens. The ELISA for detecting CA15-3 IgG is referred to here as ELISA-CA15-3, and similarly for the other ELISAs: ELISA-CEA; ELISA-CA19-9; ELISA-c-Myc; ELISA-p53; ELISA-Hsp27 and ELISA-Hsp70. The R^2^ values of mixtures of sera from controls and cancer patients exceeded 0.8 in the dilution range 1:6.25 to 1:400 for all seven ELISAs (Supplementary Figure 1), which therefore showed excellent linearity. Moreover, the inter-assay reproducibility of the ELISAs was found to be excellent (Supplementary Table 1).

**Figure 1:**
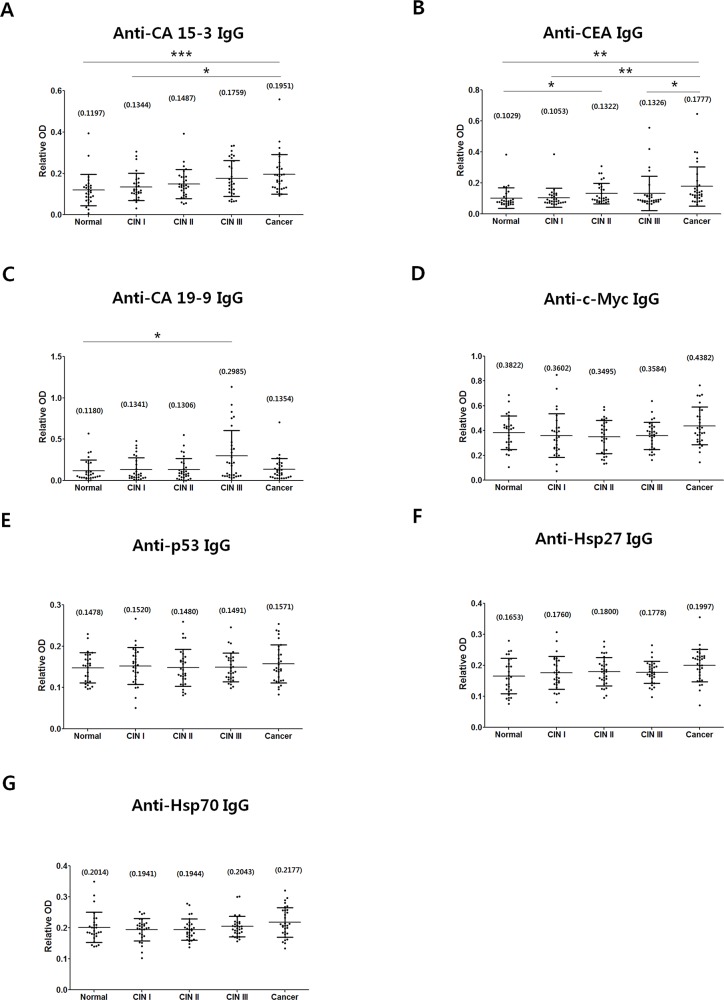
Comparison of IgG levels against CA15-3 **(A)**, CEA **(B)**, CA19-9 **(C)**, c-Myc **(D)**, p53 **(E)**, Hsp27 **(F)** and Hsp70 **(G)** were measured in normal, CIN I, CIN II, CIN III and cancer groups. The procedure for detecting IgGs against relevant TAAs by ELISA is described in Materials and Methods. Central lines are mean values, and error bars show ranges of SD values. Normal, n=28; CIN I, n=28; CIN II, n=30; CIN III, n=31; Cancer, n=31. Numbers in parenthesis are mean values.*P*-values were calculated from the Mann-Whitney-U test. The Bonferroni correction was performed, and *p*<0.05 was considered statistically significant (^*^p<0.05; ^**^p<0.01; ^***^p<0.001).

The levels of circulating IgGs against the seven TAAs are shown in Figure 1. Anti-CA15-3 IgG and anti-CEA IgG tended to increase with advancing stage of lesions (Figure 1A and 1B). Moreover, anti-CA15-3 IgG was significantly higher in the cancer group than in the normal and CIN I groups (Figure 1A). Similarly, anti-CEA IgG was significantly higher in the cancer group than in the normal, CIN I or CIN III group (Figure 1B). Unlike anti-CA15-3 and anti-CEA IgG levels, the anti-CA19-9 IgG level was only elevated in the CIN III group (Figure 1C). There were no significant differences between the various groups in terms of anti-c-Myc, anti-p53, anti-Hsp27 and anti-Hsp70 IgGs, all of which had slightly higher mean values in the cancer group than in the normal group (Figure 1D to 1G). Analysis of the differences between groups by the Bonferroni correction indicated that the anti-CA15-3 IgG and anti-CEA IgG levels were critical parameters for differentiating cervical cancer, and the anti-CA19-9 IgG level was critical for differentiating CIN III (Table 2).

**Table 2:** Differences between groups in levels of IgGs against TAAs

		CA15-3	CEA	CA19-9	c-Myc	P53	Hsp27	Hsp70
Normal vs. CIN I	*P*^a^	n.s.	n.s.	n.s.	n.s.	n.s.	n.s.	n.s.
	*P*^b^	n.s.	n.s.	n.s.	n.s.	n.s.	n.s.	n.s.
Normal vs. CIN II	*P*^a^	0.044	0.007	n.s.	n.s.	n.s.	n.s.	n.s.
	*P*^b^	0.308	**0.049**	n.s.	n.s.	n.s.	n.s.	n.s.
Normal vs. CIN III	*P*^a^	0.010	0.046	0.004	n.s.	n.s.	n.s.	n.s.
	*P*^b^	0.07	0.322	**0.028**	n.s.	n.s.	n.s.	n.s.
Normal vs. Cancer	*P*^a^	0.0001	0.0002	n.s.	n.s.	n.s.	0.030	n.s.
	*P*^b^	**0.0007**	**0.0014**	n.s.	n.s.	n.s.	0.210	n.s.
CIN I vs. CIN II	*P*^a^	n.s.	0.046	n.s.	n.s.	n.s.	n.s.	n.s.
	*P*^b^	n.s.	0.322	n.s.	n.s.	n.s.	n.s.	n.s.
CIN I vs. CIN III	*P*^a^	n.s.	n.s.	0.014	n.s.	n.s.	n.s.	n.s.
	*P*^b^	n.s.	n.s.	0.098	n.s.	n.s.	n.s.	n.s.
CIN I vs. Cancer	*P*^a^	0.002	0.0005	n.s.	0.044	n.s.	0.046	n.s.
	*P*^b^	**0.014**	**0.0035**	n.s.	0.308	n.s.	0.322	n.s.
CIN II vs. CIN III	*P*^a^	n.s.	n.s.	0.032	n.s.	n.s.	n.s.	n.s.
	*P*^b^	n.s.	n.s.	0.224	n.s.	n.s.	n.s.	n.s.
CIN II vs. Cancer	*P*^a^	0.032	n.s.	n.s.	n.s.	n.s.	n.s.	0.042
	*P*^b^	0.224	n.s.	n.s.	n.s.	n.s.	n.s.	0.294
CIN III vs. Cancer	*P*^a^	n.s.	0.004	0.033	0.046	n.s.	0.039	n.s.
	*P*^b^	n.s.	**0.028**	0.231	0.322	n.s.	0.273	n.s.

Significant differences between groups are presented as bold.

^a^ *P* value was calculated by Mann-Whitney-U test.

^b^ *P* value was calculated by Bonferroni correction, and *P*<0.05 was considered as statistical significance.

n.s.: no significant difference.

### The frequencies of samples with elevated levels of autoantibodies (IgGs) against the seven types of TAA in the normal, CIN I, CIN II, CIN III and cervical cancer groups

The proportions of samples with elevated levels of IgGs against the seven types of TAAs (Table 3) were calculated based on the IgG levels in the ELISAs (Figure 1). Samples containing levels higher than the cut-off value (95^th^ percentile) of the normal group were regarded as seropositive (Table 3). Overall the frequencies of seropositives in the CINs and cervical cancer groups appeared to be below 20%, indicating that many of their autoantibody levels overlapped with those in the normal group. Similar trends in autoantibody responses in patients with cancers have been reported previously [21, 23, 25, 34, 35]. The present results indicate that the frequencies of elevated anti-CA15-3, anti-CEA, anti-c-Myc, anti-p53 and anti-Hsp27 IgGs increase in cervical cancer, and those of anti-CEA and anti-CA19-9 IgG increase in the CIN III stage.

**Table 3:** Frequencies of autoantibodies against the CA15-3, CEA, CA19-9, c-Myc, p53, Hsp27 and Hsp70 TAAs in normal, CIN I, CIN II, CIN III and cancer groups

Group	N	CA15-3n (%)	CEAn (%)	CA19-9n (%)	c-Mycn (%)	p53n (%)	Hsp27n (%)	Hsp70n (%)
Normal	28	1 (3.6%)	1 (3.6%)	1 (3.6%)	1 (3.6%)	1 (3.6%)	1 (3.6%)	1 (3.6%)
CIN I	28	0 (0%)	1 (3.6%)	1 (3.6%)	2 (7.1%)	1 (3.6%)	2 (7.1%)	0 (0%)
CIN II	30	0 (0%)	1 (3.3%)	1 (3.3%)	0 (0%)	2 (6.7%)	1 (3.3%)	0 (0%)
CIN III	31	1 (3.2%)	3 (9.7%)	6 (19.3%)	0 (0%)	1 (3.2%)	1 (3.2%)	0 (0%)
Cancer	31	2 (6.5%)	5 (16.3%)	1 (3.2%)	4 (12.9%)	4 (12.9%)	2 (6.5%)	0 (0%)

Cut-off value for each antibody was 95^th^ percentile of normal group. *P* value was analyzed by the Fisher's exact test to compare differences between groups in the frequencies of the IgGs. *P*<0.05 was considered as significant difference. No significant differences were found between groups in the frequencies.

### Diagnostic performances of ELISA-CA15-3, ELISA-CEA, ELISA-CA19-9 and a combination of the three for discriminating the CIN I+, CIN II+, CIN III+ and cancer groups from the normal group

**Figure 2:**
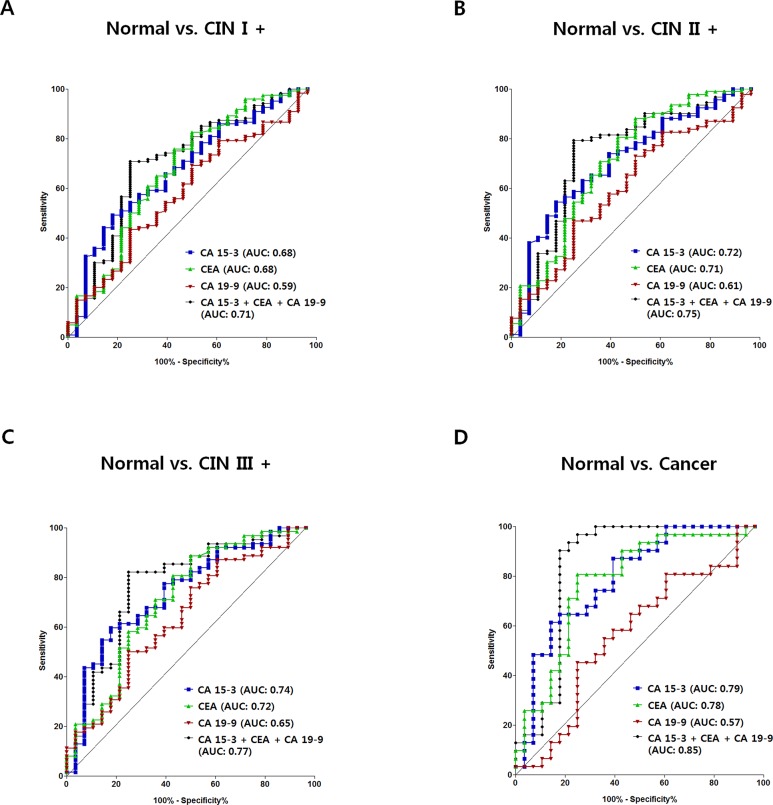
ROC curves for ELISA-CA15-3, ELISA-CEA, ELISA-CA19-9 and their combination for discriminating the CIN I+, CIN II+, CIN III+ and cancer groups from the normal group ROC curves discriminating CIN I+ from normal **(A)**, CIN II+ from normal **(B)**, CIN III+ from normal **(C)**, and cancer from normal **(D)**. Blue, anti-CA15-3 IgG; green, anti-CEA IgG; red, anti-CA19-9; black, combination.

The sensitivities, specificities, negative predictive values (NPV), positive predictive values (PPV) and accuracies of ELISA-CA15-3, ELISA-CEA, and ELISA-CA19-9 were calculated from the receiver operating characteristic (ROC) curves (Figure 2). The optimum cut-off values were obtained from the Youden's indexes of the ROC curves, which yield maximum values of sensitivity plus specificity, and the relevant diagnostic values were calculated based on these cut-off values. The three parameters (ELISA-CA 15-3, ELISA-CEA and ELISA-CA19-9) were combined by logistic regression. The individual assays had favorable sensitivities and specificities for discriminating CIN I+, CIN II+, CIN III+ and cancers from normal (Table 4). Importantly, all the area under the curve (AUC) values was elevated in the combination of ELISA-CA15-3, ELISA-CEA and ELISA-CA19-9 (Table 4). Moreover, the combination contributed to the increased sensitivity, specificity, NPV or PPV for discriminating CIN I+, CIN II+, CIN III+ and cancer from normal (Table 4).

**Table 4:** Diagnostic performances of ELISA-CA15-3, ELISA-CEA, ELISA-CA19-9 and combination assay of them for discriminating CIN I+, CIN II+, CIN III+ or cancer group from normal group

Group	Marker	AUC (95% CI)	Sensitivity	Specificity	NPV	PPV	Accuracy
Normal vs CIN I+	CA15-3	0.68 (0.56-0.78)	49.2%	82.1%	27.4%	92.2%	55.4%
	CEA	0.68 (0.54-0.78)	82.5%	50.0%	40.0%	87.6%	76.4%
	CA19-9	0.59 (0.46-0.70)	69.2%	50.0%	27.5%	85.6%	50.0%
	**Combination** CA15-3, CEA andCA19-9	0.71 (0.58-0.81)	70.8%	75.0%	37.5%	92.4%	71.6%
Normal vs CIN II+	CA15-3	0.72 (0.59-0.81)	54.4%	82.1%	35.4%	90.9%	60.8%
	CEA	0.71 (0.57-0.81)	88.0%	50.0%	56.0%	85.3%	79.2%
	CA19-9	0.61 (0.48-0.72)	72.8%	50.0%	35.9%	82.7%	67.5%
	**Combination** CA15-3, CEA andCA19-9	0.75 (0.61-0.84)	79.4%	75.0%	52.5%	91.3%	78.3%
Normal vs CIN III+	CA15-3	0.74 (0.61-0.84)	59.7%	82.1%	47.9%	88.1%	66.7%
	CEA	0.72 (0.57-0.82)	88.7%	50.0%	66.7%	79.7%	76.7%
	CA19-9	0.65 (0.50-0.75)	87.1%	39.3%	57.9%	76.1%	72.2%
	**Combination** CA15-3, CEA andCA19-9	0.77 (0.64-0.86)	82.3%	75.0%	65.6%	87.9%	80.0%
Normal vs Cancer	CA15-3	0.79 (0.64-0.88)	61.3%	85.7%	66.7%	82.6%	72.9%
	CEA	0.78 (0.63-0.88)	80.7%	75.0%	77.8%	78.1%	78.0%
	CA19-9	0.57 (0.40-0.70)	45.2%	75.0%	55.3%	66.7%	59.3%
	**Combination** CA15-3, CEA andCA19-9	0.85 (0.68-0.93)	90.3%	82.1%	88.5%	84.9%	86.4%

Three types of parameters were combined by logistic regression. Sensitivity and specificity were evaluated based on Youden's index which yields maximum values of sensitivity plus specificity. NPV, negative predictive value; PPV, positive predictive value.

Parallel and serial combinations of ELISA-CA15-3, ELISA-CEA and ELISA-CA19-9 were performed, and the sensitivities and specificities for discriminating CIN I+, CIN II+, CIN III+ or cancer from normal are presented in Supplementary Table 2. The parallel combination increased sensitivity, whereas the serial combination did not.

The increases of anti-CA15-3 and anti-CEA IgG levels in cervical cancer were marked compared to the other autoantibodies (Table 2). Therefore, the diagnostic performances of the combination of ELISA-CA15-3 and ELISA-CEA were compared to those of the combination of ELISA-CA15-3, ELISA-CEA and ELISA-CA19-9 (Supplementary Table 3). The results indicated that the combination of the two parameters was adequate for improving diagnostic performance, but that the addition of ELISA-CA19-9 was useful for improving sensitivity, specificity, NPV, PPV and accuracy.

Taken together, these results indicate that the combination assay using three types of parameters reliably discriminates CINs from normal, and powerfully discriminates cancer from normal. Our results suggest that the combination assay could be useful for primary screening of cervical cancer.

## DISCUSSION

### Suggested theories about autoantibody responses to TAAs

TAAs are thought to acquire neo-antigenicities during the development of cancers [16]. However, it is not entirely clear why the TAAs, which are fundamentally autoantigens, stimulate immune responses. Some of the hypotheses that have been suggested are as follows. First, frequent tumor cell death due to apoptosis or necrosis can lead to aberrant modification of the proteins of cancer cells that allow them to stimulate the immune systems [16, 36]. For instance, proteases released during tumor cell death can cause changes in the structural properties of the native epitopes of host proteins [16]. Second, some TAAs are inherently able to stimulate the immune system because their amino acid sequences and tertiary structures are similar to those of foreign antigens [16]. Moreover, the autoantibodies play roles in homeostasis as well as in controlling disease stage [37], and numerous types of autoantibodies are involved in such roles. Also it seems that different autoantibody responses occur in different types of cancer [38]. Characterization of these various responses may well suggest new approaches to identifying cancers.

### Autoantibody responses against TAAs in various cancers

Changes in the serum levels of anti-CA15-3, anti-CEA, anti-CA19-9, anti-c-Myc, anti-p53, anti-Hsp27 and anti-Hsp70 IgG have been previously examined as potential markers of carcinogenesis [18–26, 39]. The frequencies of these autoantibodies in various cancers, including the present data, are compared in Table 5. Direct comparison of the antibody frequencies may be inappropriate because the studies used different cut-off values and statistical criteria. However, overall the frequencies of anti-CA15-3, anti-CEA, anti-c-Myc, anti-p53 and anti-Hsp27 IgGs tended to increase in the cancers, but not those of anti-CA19-9 and Hsp70 IgG. Studies extending these findings to additional cancers are a high priority.

**Table 5:** Frequencies of autoantibodies against CA15-3, CEA, CA19-9, c-Myc, p53, Hsp27 and Hsp70 TAAs in different types of cancers

TAA	Frequency, % (number of IgG positive/number of tested)		Cancer types	Antigen used	Level change of autoantibody in cancer when compared to healthy control (Statistical significance)	Power (1-β)^b^	Cut-off (Value of normal group)	Reference
	Normal (Healthy control)	Cancer						
CA15-3 (Muc-1)	3.6% (1/28)	6.5% (2/31)	Cervical cancer	Native MUC 1	Increased (n.s.)^a^	0.94	95^th^ percentile	Our study
	23.2% (13/56)	32.8% (40/122)	Breast cancer	Recombinant MUC 1	Increased (n.s.)	N/A	Mean+3SD	[18]
	17.0% (8/47)	30.0% (6/20)	Colorectal cancer	Synthetic peptide (five MUC1 tandem repeats of the sequence PDTRPAPGSTAPPAHGVTSA)	Increased (n.s.)	N/A	Mean+2SD	[19]
CEA	3.6% (1/28)	16.3% (5/31)	Cervical cancer	Native CEA	Increased (n.s.)^a^	0.86	95^th^ percentile	Our study
	7.1% (2/28)	28.9% (15/52)	Breast cancer	Native CEA	Increased (*p*=0.025)	N/A	N/A	[20]
CA19-9 (SLeA)	3.6% (1/28)	3.2% (1/31)	Cervical cancer	Native CA19-9	No changeIncreased only in CIN III (19.3%; n.s.)^a^	Cancer (0.12) CIN III (0.88)	95^th^ percentile	Our study
	6.2% (N/A)	8.6% (N/A)	Gastrointestinal cancer	CA19-9 conjugates with PAA	No change	N/A	N/A	[39]
c-Myc	6.2% (N/A)	0% (N/A)	Breast cancer	CA19-9 conjugates with PAA	No change	N/A	N/A	[39]
	3.6% (1/28)	12.9% (4/31)	Cervical cancer	Recombinant c-Myc	Increased (n.s.)	0.42	95^th^ percentile	Our study
	0% (0/82)	18.8% (12/64)	Breast cancer	Recombinant c-Myc	Increased (*p*<0.001)	N/A	Mean+3SD	[21]
	0% (0/82)	10.7% (6/56)	Lung cancer	Recombinant c-Myc	Increased (*p*<0.001)	N/A	Mean+3SD	[21]
	0% (0/82)	15.4% (8/52)	Gastric cancer	Recombinant c-Myc	Increased (*p*<0.01)	N/A	Mean+3SD	[21]
	3.6% (1/28)	12.9% (4/31)	Cervical cancer	Recombinant p53	Increased (n.s.)	0.21	95^th^ percentile	Our study
p53	1.3% (1/76)	26% (48/182)	Breast cancer	Recombinant p53	Increased (*p*=0.0001)	N/A	2.5 times of Mean	[22]
	1.2% (2/82)	9.6% (5/52)	Colon cancer	Recombinant p53	Increased (*p*<0.05)	N/A	Mean+3SD	[23]
	8.3% (10/120)	41.7% (25/60)	Ovarian cancer	Recombinant p53	Increased (*p*<0.0001)	N/A	Mean+2SD	[24]
Hsp27	2.4% (2/82)	16.1% (9/56)	Lung cancer	Recombinant p53	Increased (*p*<0.01)	N/A	Mean+3SD	[21]
	3.6% (1/28)	6.5% (2/31)	Cervical cancer	Recombinant Hsp27	Increased (n.s.)	0.75	95^th^ percentile	Our study
	3.4% (1/29)	50.0% (17/34)	Ovariancancer	Recombinant Hsp27	Increased (N/A)	N/A	Mean+2SD	[25]
	1.9% (1/53)	37.8% (219/579)	Breast cancer	N/A	Increased (*p*<0.001)	N/A	N/A	[26]
Hsp70	3.6% (1/28)	0% (0/31)	Cervical cancer	Recombinant Hsp70	No change	0.35	95^th^ percentile	Our study
	24.1% (7/29)	13.3% (4/30)	Ovarian cancer	Recombinant Hsp70	No change	N/A	Mean+2SD	[25]
	24.1% (7/29)	32.4% (11/34)	Endometrial cancer	Recombinant Hsp70	No change	N/A	Mean+2SD	[25]
	35.9% (19/53)	40.9% (15/369)	Breast cancer	N/A	No change	N/A	N/A	[26]

ELISAs were applied for all of the studies provided to detect relevant IgGs. n.s.: no significant difference; N/A: not available.

^a^ Significant difference was found when analyzed by Mann-Whitney-U test with Bonferroni correction.

Importantly, anti-Hsp27 IgG tends to increase in cervical, ovarian and breast cancers whereas anti-Hsp70 IgG does not (Table 5). Hsps are highly conserved molecular chaperons with anti-apoptotic actions and roles in resistance to a variety of cellular stresses [40]. Expression of Hsp27, but not of Hsp70, has been reported to be inversely related to the histopathological grade of squamous cell carcinoma (oral and paraoral region) [41]. Antibody levels against Hsp27 and Hsp70 during cancer development deserve further study.

### Differences between serum TAA levels and IgG levels against TAAs

Serum CA15-3, CEA, c-Myc, p53 and Hsp27 have been observed to increase in breast cancer, CA15-3 in colorectal cancers, p53 in lung cancer and colon cancer, and Hsp27 in ovarian cancer [42–52]. Consequently, serum IgG levels against the corresponding TAAs were found at elevated levels in many studies (Table 5) [18–23]. However, some discordant cases of antibody responses have been reported. For example a negative correlation was reported between serum CA19-9 level and anti-CA19-9 IgG level (note: anti-SLeA IgG; CA19-9 is also known as SLeA [53]) in gastrointestinal cancer (Table 5) [39]. In ovarian cancer, one group found an elevated level of serum Hsp70 [54] while another detected no change in level of anti-Hsp70 IgG (Table 5) [25]. As mentioned, previous studies indicate that serum levels of CA15-3, CEA, CA19-9 and p53 increase in cervical cancer [31, 55–57] and we found that serum anti-CA15-3, anti-CEA and anti-p53 IgG levels increased while anti-CA19-9 IgG levels were unchanged (Table 5). Taken together, these findings indicate that increased levels of TAAs do not always guarantee corresponding increases in antibody responses.

### Antibody responses in cervical cancer probably involve cell surface TAAs

It has been reported that the TAAs stimulating antibody responses in breast, lung and colorectal cancer frequently include intracellular proteins such as c-Myc, p53 and Hsps [16, 58–61]. However we found weak tendency for IgG autoantibodies against intracellular TAAs to increase in cervical lesions (Table 2 and 5) whereas responses against cell surface proteins such as the mucin type antigens (CA15-3, CA19-9) and CEA were strongly affected (Figure 1) [62–65]. Therefore it is thought that cell surface antigen-related immune responses are involved in cervical carcinogenesis. It has also been suggested that the elevated level of antibody against CA15-3 (MUC-1) promotes the survival of patients with early stage ovarian, gastric, lung, pancreatic and breast cancers [59]. Moreover, there are reports that a MUC-1-based anti-cancer vaccine was effective against breast, colon and lung cancers: a vaccine made using MUC-1 as antigen elicited adaptive host immunity (B or T cell response) targeting MUC-1-expressing cancer cells, and led to cancer cell death [66–68]. The effects of vaccines against mucin-type antigens on cervical cancer merit future study.

### Changes in serum anti-CA19-9 IgG levels during cervical carcinogenesis

CA19-9 is an endothelial cell surface ligand for E-selectin that plays a major role in cancer cell adhesion, invasion and metastasis [53, 63]. Previously, it was reported that serum CA19-9 increased only in invasive cervical cancer not in the earlier CIN stages [31]. In our study, however, we found that serum anti-CA19-9 IgG was much higher in CIN III, than in the other groups (Table 2 and Figure 1C). About 40% of CIN III cases develop into invasive cervical cancer in contrast with only 1% of CIN I cases and 5% of CIN II cases [5]. The properties of CIN III cells resemble those of cancer cells and have a high potential for invasiveness [11]. Therefore it is likely that the elevated level of serum anti-CA19-9 IgG in CIN III impedes progression to cervical cancer through blocking the function of CA19-9 for the tissue invasion of cancerous cell. Since there is evidence that immunosuppression can occur during cancer development [69–72] it is possible that the drop in anti-CA19-9 IgG in cervical cancer itself is the result of immunosuppressive activity or of a requirement to increase the invasiveness of cancer cells.

### Use of an autoantibody panel to diagnose cancer

The greatest drawback of autoantibodies against TAAs for cancer screening is their low sensitivity as individual autoantibodies. Researchers have tried to overcome this drawback by using autoantibody panels. Lung cancer was detected with 76% sensitivity and 92% specificity when seven autoantibodies (anti-c-Myc, -p53, -HER-2, -Muc-1, -NY-ESO-1, -CAGE and -GBU4-4 IgGs) were combined [73], and primary breast cancer was detected with 64% sensitivity and 85% specificity with a panel of seven autoantibodies (anti-p53, -c-Myc, -HER-2, -NY-ESO-1, -BRCA1, -BRCA2 and -Muc-1 IgGs) [74]. Similarly colorectal cancer was detected with 60.9% sensitivity and 89.7% specificity with five autoantibodies (anti-p53, -p62, -c-Myc, -Imp1 and –Koc IgGs) [75].

In the present study, there were few samples with elevated autoantibody levels when cut-off values were set at the 95^th^ percentile of the normal group (Table 3). Serial combination of anti-CA15-3, anti-CEA and anti-CA19-9 ELISAs failed to improve sensitivity for discriminating CINs and cancer from the normal group, and parallel combination provided a limited improvement in sensitivity (Supplementary Table 2). Generally, both parallel and serial tests are carried out in combination assays. Each of them can in principle improve the sensitivity or the specificity of diagnosis, but neither can improve both parameters. Logistic regression can improve both sensitivity and specificity, and is based on the ROC curves obtained with the combination assay [76–80]. Indeed, satisfactory improvements in diagnostic performance were achieved in the present work when logistic regression was used (90.3% sensitivity and 82.1% specificity for discriminating cervical cancer from normal, Table 4).

### Future direction for diagnosis using autoantibodies

Numerous types of autoantibody candidates for diagnostic panels may exist in an embedded state. Therefore, the use of autoantibodies as biomarkers is likely to increase when high throughput screening systems can be used to detect novel candidates. Recently, several strategies have been developed for high throughput screening. Serological analysis of tumor antigens produced by recombinant cDNA expression cloning (SEREX), phage-display libraries, protein microarrays, serological proteome analysis (SERPA) and multiple affinity protein profiling (MAPPing) have all shown an increased ability to detect lung, breast, ovarian, prostate and liver cancers [16]. It is also likely that investigation of the expression patterns of IgG subclasses as well as of IgMs against TAAs will provide novel insights into autoantibody responses in cancers. In fact, Ann *et.al*. have reported that the pattern of expression of IgGs against MUC-1 in colorectal cancer differed from that of IgMs [19]. Such difference between IgGs and IgMs were also found in breast cancer [18].

In summary, based on previous reports and our results, the patterns of expression of autoantibodies against TAAs may be different according to the type of cancer and the stage of the lesions. Therefore, we expect that the accuracy of cancer diagnosis will be improved as knowledge of the profiles of antibodies to TAAs increases.

## MATERIALS AND METHODS

### Ethics

This study was conducted with the approval of the Institutional Review Board of EwhaWomans University Mokdong Hospital (approval No. EUMC 2016-07-067-002). All of the human samples were collected in a prospective and consecutive manner with written informed consent.

### Specimens

A total of 148 serum samples, or hysterectomy or cervical biopsy specimens were collected from women with normal cytology (n=28), CIN I (n=28), CIN II (n=30), CIN III (n=31) and cervical cancer (n=31). Each lesion was graded by three gynecology oncology specialists on the basis of review of hematoxylin and eosin (H&E)-stained sections cut from formalin-fixed and paraffin-embedded tissue blocks. Individuals with negative results in the Pap smear test, and examination of H&E-stained sections of hysterectomy specimens were classified as exhibiting normal cytology. Thus individuals in the “normal” group were those shown to have no abnormality in the cervix. Sera from the normal group were collected after examining hysterectomy specimens. Cervical cancer was graded according to the International Federation of Obstetrics and Gynecology (FIGO) staging system. Sera from the CIN I group were collected immediately after punch biopsy, and those from the CIN II and CIN III groups were collected before large loop excision of the transformation zone (LLETZ). Sera from cervical cancer patients were collected before surgery. The sera were collected from the median cubital, basilica or cephalic vein using a serum separation tube containing clot activator (8 ml, Greiner Bio-One, Australia). A clotting time of 60 min was allowed, and the samples were centrifuged at 3,000 rpm for 15 to 20 min. Finally, each serum was aliquoted into stock vials to avoid repeated freeze-thawing and stored at −80°C.

### Enzyme-linked immunosorbent assays (ELISAs)

The following seven TAAs were used to detect circulating serum autoantibodies by ELISA: native CA15-3 (Fitzgerald, USA, #30C-CP9064U), native CEA (Sigma, USA, #C4835), native CA19-9 (Fitzgerald, USA, #30-AC09), recombinant c-Myc (Fitzgerald, USA, #30R-3138), recombinant GST-tagged p53 (Enzo, USA, #BML-FW9370-0050), recombinant his-tagged Hsp27 (Fitzgerald, USA, #80R-1231) and recombinant his-tagged Hsp70 (Fitzgerald, USA, #80R-1011). 96-well ELISA plates (Greiner bio-one, Australia) were coated overnight with TAAs [50 ng for ELISA-CEA, ELISA-c-Myc, ELISA-Hsp70 and ELISA-Hsp27; 100 ng for ELISA-p53; 6 units for ELISA-CA19-9; 12 units for ELISA-CA15-3] at 4°C. The plates were blocked with 5% skim milk (Bioworld, USA) in phosphate-buffered saline containing 0.05% Tween 20 (PBST) at room temperature (RT) for 2 hrs. Then serum samples were incubated in the wells at 37°C for 90 min at optimum dilution ratios [1:12.5 for ELISA-c-Myc and ELISA-Hsp27; 1:25 for ELISA-CA15-3, ELISA-CEA, ELISA-Hsp70 and ELISA-p53; 1:50 for ELISA-CA19-9]. The optimum serum dilution ratios above were based on the linear regions of the responses as a function of serum dilution (Supplementary Figure 1). All the sera were diluted with 0.5% skim milk in PBST. Circulating serum antibodies bound to the immobilized TAAs were detected using HRP-conjugated goat anti-human IgG antibody (Sigma, USA, #A8667). The plates were washed three times with PBST between reactions, and five times prior to the reaction with substrate. Color reactions were developed using *o*-phenylenediamine (Sigma, USA), and measured at 492 nm using a FlexStation 3 Multi-Mode microplate reader (Molecular Devices, USA).

Data are presented in optical density (OD) units as employed previously [18, 20, 21, 23, 25, 81], with slight modifications. An ELISA omitting the antigen-coating step was performed to measure antibodies that react with the blocking agent (skim milk). The ODs of the seven types of ELISA were calculated from the equation: OD of ELISA with all reactants minus OD of ELISA omitting the antigen-coating step.

### Statistical analysis

Age differences between groups were analyzed by Student's t-test using Graphpad program version 5.01 (Graphpad software Inc, USA). The normality of distributions was evaluated with the Shapiro-Wilk test, and the Mann-Whitney-U test was used to evaluate differences in anti-TAA IgG levels between groups. Bonferroni corrections were performed for multiple comparisons, and *p*<0.05 was considered statistically significant. Differences between groups in the proportion of samples containing anti-TAA IgGs were analyzed by Fisher's exact test using Graphpad program version 5.01. Cut-off values for identifying seropositives were 95^th^ percentile of the normal group. The proportions of seropositives were calculated as: number of IgG positive samples/number of samples tested. Combination assays were analyzed by logistic regression using the free statistics and forecasting software, ‘Bia-reduced logistic regression version 1-1.23-r7′(http://www.wessa.net/). ROC curves and AUCs of individual or combination assays were calculated using Graphpad version 5.01 or NCSS statistical software (NCSS software, USA). The sensitivity, specificity, NPV, PPV and accuracy of each assay were determined from the ROC curves. The optimal cut-off values on the ROC curves were determined from Youden's Index which yields maximum values of sensitivity plus specificity. Power was analyzed with the G power 3.1 program (Franz Faul, Germany).

## SUPPLEMENTARY FIGURE AND TABLES


