# Supplementary material for: Use of autoantibodies against tumor-associated antigens as serum biomarkers for primary screening of cervical cancer
Source: Oncotarget. 2017 Nov 1;8(62):105425–39. doi: 10.18632/oncotarget.22231 (PMC5739648; doi:10.18632/oncotarget.22231)
Supplement: Supplementary file 1 [file oncotarget-08-105425-s001.pdf]

Use of autoantibodies against tumor-associated antigens as serum biomarkers for primary screening of cervical cancer

SUPPLEMENTARY MATERIALS

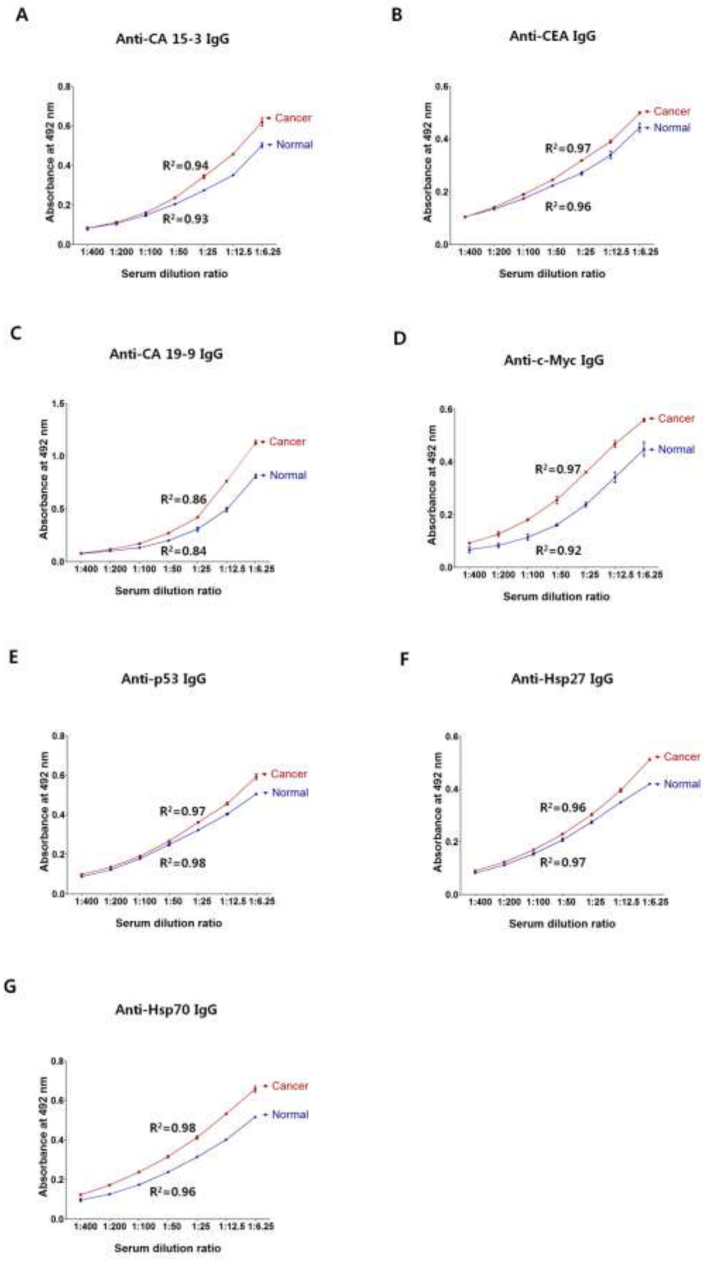

Supplementary Figure 1: Evaluating the linearity of ELISAs. Serum mixtures were prepared using 10 individual sera each from the normal and cancer groups to monitor the linearity as a function of average value. The 10 individual sera were selected randomly. 96-well plates were coated with TAAs and blocked with 5% skim milk in PBST as described in Materials and Methods. Then the serum mixtures were diluted serially (two-fold) from 1:6.25 to 1:400 and incubated on the plates at 37°C for 90 min. The subsequent steps were as described in Materials and Methods. The data represent the means ± SDs of two independent assays. Blue lines indicate the normal group, red lines the cancer group. R^2 values were used to assess the linearity of ELISA-CA15-3 **(A)**, ELISA-CEA **(B)**, ELISA-CA19-9 **(C)**, ELISA-c-Myc **(D)**, ELISA-p53 **(E)**, ELISA-Hsp27 **(F)** and ELISA-Hsp70 **(G)**.

Supplementary Table 1: Reproducibility of ELISA-CA15-3, ELISA-CEA, ELISA-CA19-9, ELISA-c-Myc, ELISA-p53, ELISA-Hsp27 and ELISA-Hsp70

Marker	Level	N	Mean	SD	CV
	High	15	0.367	0.019	5.2%
CA15-3	Medium	15	0.216	0.008	3.7%
	Low	15	0.152	0.008	5.3%
	Mean		0.245	0.011	**4.7%**
	High	15	0.361	0.019	5.3%
CEA	Medium	15	0.184	0.011	6.0%
	Low	15	0.144	0.009	6.3%
	Mean		0.230	0.013	**5.9%**
	High	15	0.520	0.009	1.7%
CA19-9	Medium	15	0.229	0.009	3.9%
	Low	15	0.118	0.005	4.2%
	Mean		0.289	0.008	**3.3%**
	High	15	0.664	0.016	2.4%
c-Myc	Medium	15	0.512	0.016	3.1%
	Low	15	0.309	0.010	3.2%
	Mean		0.495	0.014	**2.9%**
	High	15	0.285	0.013	4.6%
p53	Medium	15	0.206	0.011	5.3%
	Low	15	0.163	0.008	4.9%
	Mean		0.218	0.011	**4.9%**
	High	15	0.340	0.011	3.2%
Hsp27	Medium	15	0.237	0.007	3.0%
	Low	15	0.208	0.006	2.9%
	Mean		0.262	0.008	**3.0%**
	High	15	0.300	0.011	3.7%
Hsp70	Medium	15	0.254	0.008	3.2%
	Low	15	0.216	0.007	3.2%
	Mean		0.257	0.009	**3.4%**

The reproducibility of the ELISA was tested by evaluating interassay. Each ELISA was repeated on the different plates (triplicate) with serum samples yielding high (n=15), medium (n=15) and low (n=15) values. In this assay, noise signal by IgG reacting with blocking agent was not considered. Coefficients of variations (CVs) were calculated as standard deviations (SD) x 100/mean.

Supplementary Table 2: Diagnostic performances of parallel and serial combination assays for discriminating CIN I+, CIN II+, CIN III+ or cancer group from normal group

Group	Marker	Sensitivity	Specificity
Normal vs CIN I+	CA15-3	2.5%	96.4%
	CEA	8.3%	96.4%
	CA19-9	7.5%	96.4%
	**Parallel testing** CA15-3 or CEA or CA19-9	15.0%	89.3%
	**Serial testing** CA15-3 and CEA and CA19-9	0%	100%
Normal vs CIN II+	CA15-3	3.3%	96.4%
	CEA	9.8%	96.4%
	CA19-9	8.7%	96.4%
	**Parallel testing** CA15-3 or CEA or CA19-9	18.5%	89.3%
	**Serial testing** CA15-3 and CEA and CA19-9	0%	100%
Normal vs CIN III+	CA15-3	3.2%	96.4%
	CEA	12.9%	96.4%
	CA19-9	11.3%	96.4%
	**Parallel testing** CA15-3 or CEA or CA19-9	22.6%	89.3%
	**Serial testing** CA15-3 and CEA and CA19-9	0%	100%
Normal vs Cancer	CA15-3	6.5%	96.4%
	CEA	16.1%	96.4%
	CA19-9	3.2%	96.4%
	**Parallel testing** CA15-3 or CEA or CA19-9	19.4%	89.3%
	**Serial testing** CA15-3 and CEA and CA19-9	0%	100%

Cut-off value of each ELISA was set at 95th percentile of normal group.

Supplementary Table 3: Comparative analysis of diagnostic performances of combination assays

Group	Marker	AUC (95% CI)	Sensitivity	Specificity	NPV	PPV	Accuracy
Normal vs CIN I+	**Combination CA15-3 and CEA**	0.71 (0.57-0.81)	67.5%	75.0%	35.0%	92.1%	68.9%
	**Combination CA15-3,** CEA and CA19-9	0.71 (0.58-0.81)	70.8%	75.0%	37.5%	92.4%	71.6%
Normal vs CIN II+	**Combination CA15-3 and** CEA	0.75 (0.61-0.84)	75.0%	75.0%	47.7%	90.8%	75.0%
	**Combination CA15-3,** CEA and CA19-9	0.75 (0.61-0.84)	79.4%	75.0%	52.5%	91.3%	78.3%
Normal vs CIN III+	**Combination CA15-3 and** CEA	0.78 (0.64-0.87)	79.0%	75.0%	61.8%	87.5%	77.8%
	**Combination CA15-3,** CEA and CA19-9	0.77 (0.64-0.86)	82.3%	75.0%	65.6%	87.9%	80.0%
Normal vs Cancer	**Combination CA15-3 and** CEA	0.85 (0.69-0.93)	96.8%	75.0%	95.5%	81.1%	86.4%
	**Combination CA15-3,** CEA and CA19-9	0.85 (0.68-0.93)	90.3%	82.1%	88.5%	84.9%	86.4%

Table shows AUC value, sensitivity, specificity, NPV, PPV and accuracy when two parameters (anti-CA15-3 IgG and anti-CEA IgG) or three parameters (anti-CA15-3 IgG, anti-CEA IgG and anti-CA19-9 IgG) were combined. The parameters were combined by logistic regression, and cut-off values were determined from Youden's Index which elicits maximum sum of sensitivity and specificity.
